# Addressing Stroke Signs and Symptoms Through Public Education: The Stroke Heroes Act FAST Campaign

**Published:** 2008-03-15

**Authors:** Hilary K Wall, Brianne M Beagan, H June O'Neill, Kathleen M Foell, Cynthia L Boddie-Willis

**Affiliations:** Massachusetts Department of Public Health; Massachusetts Department of Public Health, Boston, Massachusetts; Massachusetts Department of Public Health, Boston, Massachusetts; Massachusetts Department of Public Health, Boston, Massachusetts; Massachusetts Department of Public Health, Boston, Massachusetts

## Abstract

**Introduction:**

In 2003, only 18% of Massachusetts adults were aware of all signs and symptoms of stroke, but 80% would call 9-1-1 if they thought someone was having a stroke or heart attack. Because early recognition leads to early treatment and improved clinical outcomes, increasing symptom recognition could have an impact on stroke survival and stroke patients' quality of life.

**Methods:**

We conducted secondary research to identify messages with evidence-based effectiveness for communicating stroke signs and symptoms. From these results, a Stroke Heroes Act FAST animation was created and concept-tested. Non-Hispanic white and non-Hispanic black women aged 40 to 64 years received education on stroke signs and symptoms. Knowledge change about stroke signs and symptoms was calculated immediately following and 3 months after the education session.

**Results:**

Using Stroke Heroes Act FAST educational materials that were developed, 72 women (mean age, 54 years; 15.5% were non-Hispanic blacks) received education about signs and symptoms of stroke and took the pretests and posttests to assess knowledge change. Immediately after the education session, significant increases were seen in the percentage of participants who recognized that facial droop (92% vs 99%, *P* = .02) and arm weakness or numbness (86% vs 97%, *P* = .004) were symptoms of stroke. Of the 65 participants who were given the 3-month follow-up survey, 100% remembered slurred speech and facial drooping as symptoms; 98.5% recalled arm weakness or numbness; and 97% would call 9-1-1 if they thought someone was having a stroke. None of these is a significant change from the posttest.

**Conclusion:**

The Stroke Heroes Act FAST kit may be a useful tool for improving knowledge of stroke signs and symptoms among adults.

## Introduction

Stroke is the third leading cause of death in the United States (1), and it is the third leading cause of death in Massachusetts (2). A national *Healthy People 2010* (*HP 2010*) goal is to reduce stroke deaths to 48 per 100,000 people (3). In 2004, 43 per 100,000 Massachusetts residents died from stroke, whereas 50 per 100,000 people nationwide died from stroke (1). Moreover, in 2005, 3% of Massachusetts adults reported having had a stroke (4). Although the state's stroke death rate meets the *HP 2010* target, unpublished state vital records data indicate that deaths from stroke in Massachusetts have not significantly decreased since 1994. Several communities have disproportionately high rates of stroke risk factors, prevalence, and death.

In June 1998, the Brain Attack Coalition, a group of national professional, volunteer, and government entities dedicated to reducing stroke-related death and disability, reached consensus on the symptoms of stroke. Previously, standardized definitions for stroke signs and symptoms did not exist. The consensus symptoms are sudden numbness or weakness of face, arm, or leg, especially on one side of the body; sudden confusion or trouble speaking or understanding speech; sudden trouble seeing in one or both eyes; sudden trouble walking, dizziness, or loss of balance or coordination; and sudden severe headache with no known cause (5). These "suddens" are commonly used to convey stroke symptoms in clinical and public health settings and among advocacy organizations concerned with stroke.

The "suddens" were adopted by several national and state-based educational campaigns. Since consensus was reached, Massachusetts advocacy organizations have annually conducted at least one campaign on the signs and symptoms of stroke. Yet in 2003, only 18% of Massachusetts adults were aware of all signs and symptoms of stroke (6), but 80% would call 9-1-1 if they thought someone was having a stroke or heart attack (6). Because early recognition leads to early treatment and improved clinical outcomes, increasing symptom recognition could vastly improve stroke survival and quality of life (7).

In March 2004, the Massachusetts Public Health Council approved a primary stroke service (PSS) hospital designation (8). This state-level designation is conferred by the Massachusetts Department of Public Health (MDPH), Division of Health Care Quality. Each hospital with this designation meets mandated criteria based on guidelines for diagnosing and treating acute stroke patients. To address the lack of recognition of stroke symptoms in Massachusetts, the MDPH Heart Disease and Stroke Prevention and Control Program (HSPC) successfully advocated for the inclusion of community education on stroke signs and symptoms in the PSS regulations.

Prior short-term studies have demonstrated that public education campaigns can increase stroke recognition (9-12) and the quickness with which stroke patients seek and receive appropriate care (9,13). To support Massachusetts hospitals in meeting the PSS community education requirements, HSPC began in July 2004 to explore effective stroke symptom messages that were evidence-based and could be incorporated into a long-term, comprehensive public education campaign. We present here the results of this effort.

## Methods

By combining state-appropriated funds for stroke education and federal funds from the Centers for Disease Control and Prevention, HSPC is implementing a comprehensive, evidence-based public education campaign. An analysis of stroke prevalence, mortality, and hospitalization surveillance data by race/ethnicity, age, and sex indicated that the initial priority population for the comprehensive campaign was English-speaking women who were either non-Hispanic white or non-Hispanic black and the potential caregivers of these women. To guide stroke education in Massachusetts, a Stroke Signs and Symptoms Advisory Group was formed comprising health professionals, stroke survivors, and representatives from communities in which the risk for stroke, heart disease, or chronic disease is high.

### Secondary research

A social marketing and communications company, Geovision, Inc, was hired to conduct secondary research on existing messages about stroke signs and symptoms. It conducted a comprehensive literature search and informal inventory of other unpublished sources. The results were used to develop an evidence-based approach to educating the lay public to recognize the signs of stroke and respond by calling 9-1-1. The search for messages and materials included online investigation and direct inquiry to national and international medical, public health, academic, and advocacy agencies and organizations that promote awareness, prevention, and treatment of stroke. These organizations included state and international health departments, hospitals and research centers, medical foundations, and universities. The search also included an assessment of barriers to calling 9-1-1 in response to acute medical events among the priority populations living in communities where the risk for stroke is elevated. Existing knowledge and strategies relating to the effective placement of health communication materials about medical emergencies were also assessed in the literature search.

### Concept testing

Using results from the secondary research, Geovision created a 3-minute animation to teach the signs of stroke. We convened five groups to test the concept of the animation: three mixed-audience groups, one group of stroke educators, and one group of stroke survivors. All participants received financial compensation for attending a 90-minute session. We created three separate discussion guides to solicit comments on the animation concept and information content. Immediately after viewing the animation and before any discussion, the mixed groups completed a brief survey to evaluate information recall and message resonance.

The three mixed-audience groups consisted of 1) non-Hispanic white and non-Hispanic black men and women aged 63 or older who had not had a stroke; 2) non-Hispanic white and non-Hispanic black adult children of parents aged 65 or older; and 3) providers of community elder programs or non-health–care services. Because of high prevalence of stroke and stroke-related hospitalization and mortality in Fall River, Lowell, and Springfield, residents in these communities were recruited for the mixed-audience groups by professional telephone recruiters and community-based organizations. Participants provided information including age, household income levels, and employment status.

Twelve known stroke educators were contacted to participate in the stroke educators concept testing group. Additionally, education staff from over 40 hospitals and health care organizations were solicited to participate in the stroke educators group. Stroke survivors were recruited from a pool provided by MDPH, the Mt. Auburn Hospital Stroke Service Program, and the Watertown Senior Center.

### Retention pilot

Risk of stroke is higher for older and low-income women, and the rate of death from stroke more than triples with each added decade of life after the age of 45 (1). To assess message retention from our public education model, we conducted a pilot project with several cardiovascular disease risk reduction educators from the Well-Integrated Screening and Evaluation for Women Across the Nation (WISEWOMAN) program from the MDPH Women's Health Network. The network provides free breast and cervical cancer screening and diagnostic services to low-income women aged 40 to 64. Sites selected to participate in WISEWOMAN provide heart disease and stroke prevention services that include cardiovascular health risk assessment and lifestyle interventions by the cardiovascular risk reduction educators. The project proposal was submitted to and approved by the MDPH Human Research Review Committee. HSPC trained the educators, who in turn taught stroke signs and symptoms to a group of Women's Health Network clients.

Participants were asked to sign an informed consent form and provide basic demographic information. To assess changes in knowledge of stroke signs and symptoms, participants were given a pretest and posttest immediately before and after the education session. Participants received a $10 grocery store gift card for attending the session. Three months later, a follow-up telephone survey was conducted, after which participants received a $15 grocery store gift card.

Data were entered into Excel 2003 (Microsoft Corporation, Redmond, Washington) and then exported to SAS (SAS Institute, Inc, Cary, North Carolina) for analysis. Descriptive statistics were determined for demographic variables. Paired *t*-tests were used to determine differences in mean between pretest and posttest results as well as posttest and 3-month follow-up results. We considered the results of the paired *t*-tests statistically significant when the corresponding *P* value was ≤.05.

## Results

### Secondary research

Geovision identified 24 private organizations and 20 national health departments and contacted them for sample materials. Three departments provided relevant program information and materials. Thirty articles were reviewed and summarized.

The search revealed that a comprehensive public awareness campaign that includes mass media can increase stroke recognition but should target family, coworkers, and caregivers of those at highest risk for stroke. Moreover, educational efforts should focus on behaviors that promote early seeking of hospital care.

The research, however, yielded a paucity of stroke education messages with evidence-based effectiveness. Among them was the Cincinnati Prehospital Stroke Scale (CPSS) (14), a three-item scale based on a simplification of the National Institutes of Health Stroke Scale (15), which has high sensitivity and specificity for identifying stroke patients who are candidates for thrombolytic treatment when performed by a physician (15) and has similar results when used by prehospital care providers (14). Moreover, the CPSS has been accurately administered by untrained laypeople to identify stroke signs in mock patients (16) and in stroke survivors (17) when prompted by a 9-1-1 telecommunicator. On further investigation, we learned that the CPSS had been successfully modified by adding a fourth item, so that it could be used by laypeople before they called 9-1-1. This item provided instructions for laypeople to call 9-1-1 if they observed stroke signs in someone. Kleindorfer et al created the FAST acronym for lay assessment of stroke symptoms (Table 1) (18). During a retrospective chart review of 3500 stroke patients, the authors found that the FAST message identified 88.9% of patients with stroke or transient ischemic attack (TIA) (i.e., brief stroke-like symptoms that can occur with no lasting damage) (18).

On the basis of its findings from the secondary research and on knowledge of basic memory techniques (e.g., mnemonics or acronyms, repetition, rhythm or songs), Geovision recommended creating a 3-minute musical animation using the FAST acronym. The animation was concept-tested and produced for English speakers.

### Concept testing

Of 41 people recruited for the three mixed-audience groups, 34 participated. The mean age of participants was 58.1 years (Table 2), and they included seniors (41%), adults with senior parents (38%), and health care workers who care for seniors (21%). Twenty-two participants (65%) were female, and 23 (68%) were non-Hispanic whites. Forty-four percent of participants earned less than $20,000 per year.

Immediately after being shown the 3-minute animation, participants completed a brief form to assess information recall. From the three groups, 100% of participants indicated that the topic of the video was stroke and identified the action to take at the first sign of a stroke (call 9-1-1); 76.5% recalled the three symptom components of FAST (Face, Arm, and Speech).

Of the seven stroke survivors who participated in concept testing, three (43%) were female and all were non-Hispanic white (Table 3). The mean age was 54.1 years. Four (57%) had had ischemic strokes (i.e., strokes caused by a blocked blood vessel in the brain).

Nine stroke educators were part of the final concept-testing group. All nine were non-Hispanic white women, and they represented seven different hospitals or health care organizations. Their titles were diverse and included neuroscience educator and director of rehabilitation services.

Participants from all five groups thought the animation provided a comfortable, upbeat, "less scary" approach to a serious health issue, and that the animation and music were simple, direct, and uncluttered. Participants consistently described the accompanying song as "catchy" and the animation style as simple but appealing. Recommendations included creating a visual reinforcement of the FAST checklist, depicting a younger character in at least one of the scenarios, and accentuating the distorted speech bubble. Some stroke educators expressed concern about showing only the three most common symptoms without suggesting that other symptoms may indicate stroke.

**Figure.**
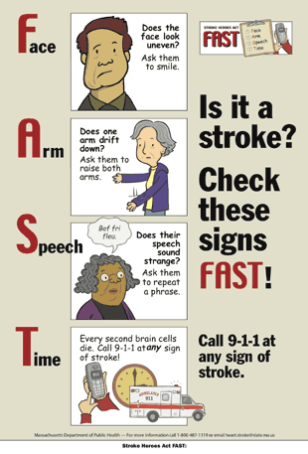
Stroke Heroes Act FAST poster

We incorporated suggestions from the concept testing into a final animation and created a brochure and poster (Figure) using the characters from the animation. These pieces were included in a Stroke Heroes Act FAST educational kit, which had instructive PowerPoint presentations with information about stroke signs, symptoms, and risk factors; an educator's guide with strategies for effectively reaching lay audiences; a press release; and evaluation forms. The kit, which we designed as a train-the-trainer module, is available through HSPC.

### Retention pilot

Seventy-two women participated in the retention pilot. One participant refused to provide demographic information. The mean age of participants was 53.7. Participants were primarily non-Hispanic whites (76%) and had never had a stroke (99%). Almost half (49%) of participants had a family history of stroke (Table 4).

Immediately after the education session, we saw significant increases in the percentage of participants who recognized that facial droop (91.7% vs 98.6%, *P* = .02) and arm weakness or numbness (86.1% vs 97.2%, *P* = .004) were symptoms of stroke. Moreover, we also saw significant increases in the percentage of participants who thought chest pain and shortness of breath were not stroke symptoms. The percentage of participants who recognized "call 9-1-1" as the action to take after observing stroke symptoms also increased significantly, from 81.9% to 98.6% (*P* < .001) (Table 5).

Three months after the education session, we conducted a follow-up survey of the participants. Seven participants could not be reached. Of the 65 participants who were given the survey, 100% remembered slurred speech and facial drooping as symptoms of stroke; 98.5% remembered arm weakness or numbness; 96.9% would call 9-1-1 if they thought someone was having a stroke. None of these is a significant change from the posttest (Table 6). However, significantly fewer participants remembered correctly that chest pain (87.7% vs 55.4%, *P* < .001) and shortness of breath (84.6% vs 50.8%, *P* < .001) were not symptoms of stroke. Moreover, the percentage of participants who recognized all components of the FAST acronym declined significantly, from 100.0% to 78.5% (*P* < .001).

## Discussion

Concept testing of the Stroke Heroes Act FAST animation yielded positive results for message recall and aesthetics. Despite concerns from stroke educators during concept testing that FAST does not convey all the signs and symptoms of stroke, Kleindorfer et al demonstrate that the FAST acronym successfully identified 88.9% of stroke patients (18).

In this assessment, we found that among women aged 45 to 64, using the FAST message through the Stroke Heroes Act FAST kit is an effective way to facilitate short-term knowledge change and message retention about stroke signs and symptoms. Although the scientific literature lacks messages that successfully affect knowledge of stroke signs and symptoms, the FAST acronym shows promise as an effective memory device and message, especially when coupled with the colorful animation and catchy tune in the Stroke Heroes Act FAST animation and accompanying materials. As Kleindorfer et al conclude, however, whether the FAST message is easier to recall than the "suddens" has yet to be determined (18).

Relevant changes from pretest to posttest include the significant increases in participants who recognized that facial droop and arm weakness or numbness are symptoms of stroke, who would call 9-1-1 if they thought someone was having a stroke, and who learned that the presence of only one symptom is enough to suspect someone is having a stroke. The ability of the Stroke Heroes Act FAST kit to increase awareness of the latter two concepts could increase the acceptance of stroke as an emergency condition and lead to more prompt treatment and decreased death and disability from stroke.

Stern et al observed a significant gain in stroke knowledge only immediately after an educational program was administered (11). Unlike Stern et al, however, we were able to conduct a follow-up assessment, and we found no significant differences from the posttest to 3-month follow-up in the percentage of participants who recognized slurred speech, drooping of the face, and arm weakness or numbness as signs of stroke. However, almost half of participants had difficulty recalling that chest pain and shortness of breath were not stroke signs. These results suggest that participants were confident in knowing the true signs of stroke but less so in ruling out false positives. However, because chest pain and shortness of breath are major signs of heart attack and almost all participants would call 9-1-1 if they thought someone was having a stroke independent of symptom presentation, a person suffering from these symptoms would still receive quick emergency response and care even if their condition was incorrectly identified as stroke.

We noted some decrease in message retention after 3 months. The percentage of participants who recognized all components of the FAST acronym significantly declined from 100.0% to 78.5%. Although this retention rate is high, it suggests that education on stroke signs and symptoms should be conducted periodically or perhaps enhanced with a media campaign. Consequently, the Stroke Heroes Act FAST animation became the basis for an English-language mass media campaign, including television public service announcements, transit card placards for subway and buses, and newspaper advertisements. This campaign is being evaluated. The FAST message also will be culturally and linguistically adapted for Spanish, Portuguese, and Asian audiences to educate the largest populations of non-English speakers in Massachusetts.

The main limitation of our analysis is the lack of power and sample size calculation. As a result, observed results may be due to chance. Future formal studies should be conducted with larger samples. Another limitation is that, because this was intended as a pilot project, we used a convenience sample and, as a result, had more than two times the percentage of black, non-Hispanic women than is found in the overall Massachusetts population. Lastly, in our review of pertinent literature on stroke signs and symptoms education, we found that investigators rarely specify the exact wording of the messaging used to change knowledge. We hope that in the future, investigators will provide more detail about their education campaigns so that we can collectively evaluate the success of these messages and develop better ones.

In our analysis, we have yet to assess whether the change in knowledge and message retention has resulted in improved time from stroke symptom onset to treatment. Attributing any changes in stroke patients' arrival time at hospitals solely to the Stroke Heroes Act FAST campaign and not to secular changes may be challenging.

Although national leaders in the treatment of stroke promote the use of the "suddens" for stroke signs and symptoms, to our knowledge this device was developed by consensus and not tested for retention or ability to change knowledge. The findings show that the Stroke Heroes Act FAST animation, which uses the FAST acronym and message, might be a useful tool for improving knowledge of stroke signs and symptoms among adults.

## Figures and Tables

**Table 1:** FAST Acronym, Based on the Cincinnati Prehospital Stroke Scale

**Component**	**Description**
**F**ace	Face numbness or weakness, especially on one side
**A**rm	Arm numbness or weakness, especially on one side of body
**S**peech	Slurred speech or difficulty speaking or understanding
**T**ime	Time to call 9-1-1 if these signs occur suddenly or are accompanied by the loss of vision, the loss of balance with dizziness, or the worst headache of your life, with no known cause, both sudden and severe

Source: Reference 18.

**Table 2:** Demographic Characteristics of the Mixed-Audience Concept Testing Group (N = 34), Stroke Heroes Act FAST Project, March 2005

**Characteristic**	Mixed audience
**Age, y**	
Mean (standard deviation)	58.1 (13.3)
**Sex (%)**	
Female	22 (65)
**Race (%)**	
Non-Hispanic white	23 (68)
Non-Hispanic black	11 (32)
**Recruitment criteria (%)**	
Seniors	14 (41)
Adults with senior parents	13 (38)
Health care workers	7 (21)
**Income^a^ (%)**	
<$10,000	8 (24)
$10,000 - $19,999	7 (21)
$20,000 - $34,999	3 (9)
$35,000 - $49,999	3 (9)
$50,000 - $64,999	2 (6)
$65,000 - $84,999	2 (6)
>$85,000	3 (9)
**Residence (%)**	
Fall River area	8 (24)
Lowell area	12 (35)
Springfield area	14 (41)

a Six of the 34 participants did not provide income data.

**Table 3:** Demographic Characteristics of the Concept Testing Group of Stroke Survivors (N = 7), Stroke Heroes Act FAST Project, March 2005

**Characteristic**	Stroke survivors
**Age, y**	
Mean (standard deviation)	54.1 (12)
**Sex (%)**	
Female	3 (43)
**Race (%)**	
Non-Hispanic white	7 (100)
**Stroke Type (%)**	
Ischemic	4 (57)
Hemorrhagic	2 (29)
Other	1 (14)

**Table 4:** Demographic Characteristics (N = 71) of Participants in the Pilot for Message Retention, Stroke Heroes Act FAST Project, October–November 2005

**Characteristic**	Baseline^a^
**Age, y**	
Mean (standard deviation)	53.7 (8)
**Sex (%)**	
Female	71 (100)
**Race/ethnicity (%)**	
Non-Hispanic white	54 (76)
Non-Hispanic black	11 (15)
Hispanic	5 (7)
Asian	0
Other	1 (1)
**Ever had a stroke (%)**	
Yes	0
No	70 (99)
Don't know	1 (1)
**Family history of stroke (%)**	
Yes	35 (49)
No	35 (49)
Don't know	1 (1)

a One participant did not complete demographic information.

**Table 5:** Pretest and Posttest Survey Results (N = 72) in the Pilot for Message Retention, Stroke Heroes Act FAST Project, October–November 2005

**Question and Answer Choices**	Pretest (%)	Posttest (%)	*P* value
**1. Which of the following do you think is a symptom of stroke (check all that apply)**			
Slurred speech	94.4	98.6	.08
Drooping of the face	91.7	98.6^b^	.02
Chest pain^a^	70.8	84.7^b^	.007
Arm weakness or numbness	86.1	97.2^b^	.004
Shortness of breath^a^	62.5	81.9^b^	.001
*% that correctly identified the 3 true symptoms*	76.4	94.4^b^	<.001
*% that correctly identified whether all 5 were symptoms*	44.4	76.4^b^	<.001
**2. If you thought someone was having a stroke, what is the first thing you would do? (Check only one)**			
Take them to the hospital	12.5	0.0	NA^d^
Tell them to call their doctor	0.0	0.0	NA^d^
Call 9-1-1	81.9	98.6^b^	<.001
Call their spouse or a family member	0.0	0.0	NA^d^
Tell them to lie down	5.6	1.4	NA^d^
**3. How many stroke symptoms must a person have before you would do something? (Check only one)**			
All of them	6.9	2.8	NA^d^
One	77.8	94.4^b^	.002
Two or more	15.3	2.8	NA^d^
**4. If a person has a stroke and gets treatment quickly they will most likely (Check only one)**			
Die from the stroke	0.0	0.0	NA^d^
Have severe physical disability	1.4	2.8	.57
Be able to recover with little or no disability	95.8	95.8	NA^d^
Not be able to speak	0.0	0.0	NA^d^
Did not respond	1.4	1.4	NA^d^
*Multiple answer of severe disability and recover^e^*	1.4	0.0	NA^d^
**5. When you think about stroke signs and symptoms, what does FAST stand for?**			
Face	NA^c^	100.0	NA^d^
Arm	NA^c^	100.0	NA^d^
Speech	NA^c^	98.6	NA^d^
Time	NA^c^	100.0	NA^d^
*% that correctly identified all 4 items*	NA^c^	98.6	NA^d^

NA indicates not applicable.

a Percentages indicate the proportion of participants who correctly answered that this is not a stroke symptom.

b Change from pretest to posttest is statistically significant at *P* < .05.

c The question was not asked during pretest.

d *P* value was not calculated.

e One respondent chose two possible answers.

**Table 6:** Survey Results (N = 65) of Posttest and 3-Month Follow-up in the Pilot for Message Retention, Stroke Heroes Act FAST Project, January–February 2006

**Question/answer choices**	Posttest (%)	3-month follow-up (%)	*P* value
**1. Which of the following do you think is a symptom of stroke? (check all that apply)**			
Slurred speech	100.0	100.0	NA^c^
Drooping of the face	100.0	100.0	NA^c^
Chest pain^a^	87.7	55.4^b^	<.001
Arm weakness or numbness	96.9	98.5	.57
Shortness of breath^a^	84.6	50.8^b^	<.001
*% that correctly identified the 3 true symptoms*	96.9^b^	98.5	.57
*% that correctly identified whether all 5 were symptoms*	80.0^b^	47.7^b^	<.001
**2. If you thought someone was having a stroke, what is the first thing you would do? (Check only one)**			
Take them to the hospital	0.0	3.1	NA^c^
Tell them to call their doctor	0.0	0.0	NA^c^
Call 9-1-1	100.0	96.9	.16
Call their spouse or a family member	0.0	0.0	NA^c^
Tell them to lie down	0.0	0.0	NA^c^
**3. How many stroke symptoms must a person have before you would do something? (Check only one)**			
All of them	1.5	4.6	NA^c^
One	96.9	90.8	.16
Two or more	1.5	4.6	NA^c^
**4. If a person has a stroke and gets treatment quickly they will most likely (Check only one)**			
Die from the stroke	0.0	0.0	NA^c^
Have severe physical disability	3.1	3.1	NA^c^
Be able to recover with little or no disability	96.9	96.9	NA^c^
Not be able to speak	0.0	0.0	NA^c^
Did not respond	0.0	0.0	NA^c^
**5. When you think about stroke signs and symptoms, what does FAST stand for?**			
Face	100.0	90.8^b^	.01
Arm	100.0	93.8^b^	.04
Speech	100.0	86.1^b^	.002
Time	100.0	81.5^b^	<.001
*% that correctly identified all 4 items*	100.0	78.5^b^	<.001

NA indicates not applicable.

a Percentages indicate the proportion of participants who correctly answered that this is not a stroke symptom.

b Change from posttest to 3-month follow-up is statistically significant at *P* < .05.

c *P* value was not calculated.
